# Physiological Candles

**Published:** 1889-12-07

**Authors:** 


### The Doctor's Pulpit.

### PHYSIOLOGICAL CANDLES.

### The Moral Fruits of Darwinism.

The wolf is a successful hunter, because nature has endowed him with a keen and eager appetite, unusual powers of limb and teeth, and a relentless disposition. There can be no doubt that at the moment of seizing his prey he is impelled by a natural ferocity which is absolutely and entirely irresistible. A man in a similar state of feeling would be mad, and altogether irresponsible for his actions. What would be madness in the man is the natural condition of the wolf at the moment of supreme hunger. The animal is blind, deaf, and insensible to every conceivable consideration except that of seizing and holding the prey.

It cannot be questioned that a disposition of this kind has survived in human creatures even up to the present time. The reason why a man is successful in securing the excessive indulgence of animal passions, in the gaining of wealth, in the acquisition of certain coveted positions among his fellows, is not necessarily that he possesses the all-round attributes of man in higher perfection than his fellows. In most cases it is because the predominating element of his character is lust, hungry greed, an insatiate desire for pre-eminence which he cannot resist. Why do some men who were born poor get rich in spite of every obstacle? It is because they are men of superior ability, say some; or because they are extremely lucky, say others, and with much less reason. Nothing of the kind. In most cases it is because the money-hunger is as keen and as fierce in them as the flesh-hunger is in the wolf, and also because the other and nobler attributes of man, such as regard for the interests of their fellow-men, practical sympathy with their necessities, a clear perception and regard for justice, and the like, are deficient or altogether wanting. From some persons, indeed, these higher attributes of humanity are almost as completely absent as from the wolf. Many a bleating and helpless lamb would be spared, one thinks, if the wolf had but a little pity; many a child who wanders from his village and is overtaken by the night would escape being devoured if the animal, even though very hungry, had but imagination enough to picture to himself the despair of the unhappy mother at home. But that is impossible. A wolf never spares. He cannot spare. Nature has denied him every faculty which would interfere with, and in any way restrain, the one predominating characteristic of flesh-hunger.

It has been usual to approach the man in whom the wolf-like faculties have survived in extraordinary measure from the moral side only, and to denounce him utterly. That, the writer is persuaded, is to a large extent futile. You might just as well denounce a wolf. The animal is quite indifferent to your denunciations, and he cannot help being so. There is, however, this difference between the man and the wolf—that the former, though cursed with such an animal survival as blind and vulgar greed, has other faculties, which, even if small and ill-developed, have yet an actual existence in him, and a potentiality of education. These faculties may, under favourable circumstances, be utilised to restore the balance, and to correct and restrain the excesses of the lower desires.

The practical thing to do is to recognise that men and women are animals, with mental and moral faculties; and that the present stage of some is not one even of manlike, much less of divine perfection; but one of slow and painful, though certainly progressive development. The order is from the animal, through the man, and towards the divine ideal. Mr. Matthew Arnold, from his purely intellectual and critical standpoint, saw a "tendency" in men "making for righteousness;" and as "righteousness" was his standard of perfection, he clearly believed that the ultimate destiny of man was at least completely antipodal to his wolf-like beginnings. Mr. Darwin, whose intellect was as clear as Arnold's, and whose knowledge and opportunities were of a totally different order, arrived practically at the same conclusion. St. Paul, whose visual standpoint was as far removed from either of theirs as it could well be, and who had the disadvantage of living eighteen centuries earlier, beheld "the whole world groaning and travelling in pain together," waiting for what, in expressive phrase, he called the "redemption of the body," or, as we might now say, waiting for the complete subjugation of the lower and baser appetites of the mere animal, and the prevalence of pure intellectuality, morality, and sympathy. All these three typical men, occupying typical and entirely distinct standpoints, arrived practically at the same conclusion with regard to the nature and progressive destiny of man. We may venture to accept their conclusions as, on the whole, true and sound, and, above all, as forming an adequate basis for practical effort.

To return to our man who is physiologically so much more akin to the wolf in character than to his bipedal and more intellectual and moral *confrères*. What is to be done to him and with him? For in any rational society we must do something to him as well as with him. Means must be found so to check and moderate his money-seizing propensities as at least to prevent his doing injuries to others. That is an axiom. In private life, every individual person who deals with him must be specially on watch and guard, remembering that, being of a wolfish nature, he is always ready to take every sort of advantage, whether fair or unfair; remembering also, that the moral and intellectual checks which are sufficient to restrain other people are to a very large extent inoperative with him. In the interests of society and of less wolf-like individuals than himself it is clear that he must be restrained.

But the question we wish to emphasise now and to answer is rather—What can be done *with* the wolf-like man, than—What can be done *to* him? It may be answered by asking another—Why not approach him from the scientific instead of the moral side? If moral denunciation be your only weapon, you merely succeed in putting him on the defensive. Like his lupine prototype, the whole energy of his mind, such as it is, is concentrated on the one idea of successful defence and escape. But if you approach him scientifically, with the truce-flag of instruction instead of the horsewhip of moral denunciation, you at least get at the faculties of attention and consideration, which, in common with all other human beings, he undoubtedly possesses; and you also get at the other human attributes of justice, sympathy, and altruism, which, though in a small and dormant degree, he, in common with higher human beings, almost certainly possesses. If you fail, like the ancient mariner with the wedding guest, to hold him with the "skinny hand" of moral hatred, it is just possible that, like him, you may hold him with the "glittering eye" of a cold and impartial science.

Here is one of the most valuable moral fruits of Darwinism, that it can substitute, on occasions, the neutral tint of scientific impartiality for the flaming red of virtuous indignation. Give your wolfman a comprehensive and comparative view of human and animal nature. Teach him to understand that the resistless flesh-hunger of the wolf, though natural and inevitable in it, is not natural to the higher animal man, at any rate at his present stage of development; but is a low, cunning, and irrational survival from animal times, and as anachronous and absurd in a civilised man as a fig-leaf or war paint. The too-greedy and self-asserting man is, in short, a curiosity, a museum specimen, although of course a dangerous one. It is quite possible that if the habit of treating him as such were persistently and generally practised, he might in time come to have a glimmering of the actual scientific aspect of the case, and thus gradually develop an effectual desire to crucify the wolf in order that the man might live.

